# P-1439. Evaluation of Mpox Knowledge, Attitude, and Willingness to Accept Mpox Vaccine among People living with HIV and Men who Have Sex with Men in Rivers State, Nigeria

**DOI:** 10.1093/ofid/ofaf695.1626

**Published:** 2026-01-11

**Authors:** Chizaram Onyeaghala, Ifeoma Ugboma, Nelson Oruh, Vivian Ogbonna

**Affiliations:** UPTH, Port Harcourt, Rivers, Nigeria; UPTH, Port Harcourt, Rivers, Nigeria; International Advancement for Humanity, Port Harcourt, Rivers, Nigeria; UPTH, Port Harcourt, Rivers, Nigeria

## Abstract

**Background:**

There is a paucity of information regarding mpox-related knowledge, risk perception, and vaccine acceptance among people living with human immunodeficiency virus (PLHIV) and men who have sex with men (MSM), a high-risk population for mpox, in countries with intersecting epidemics of HIV and mpox, such as Nigeria.

**Figure:**
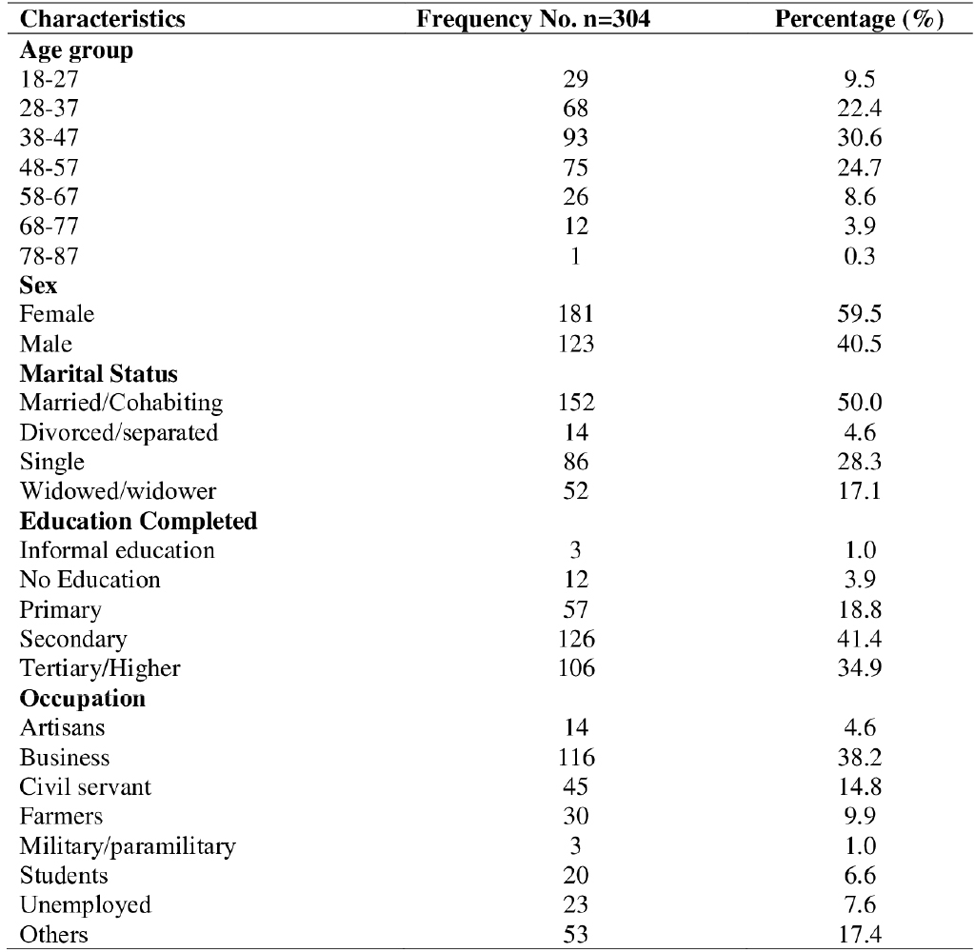
Socccth HIV and Men who have sex with Men in selected health facilities in Rivers State Nigeria

**Figure:**
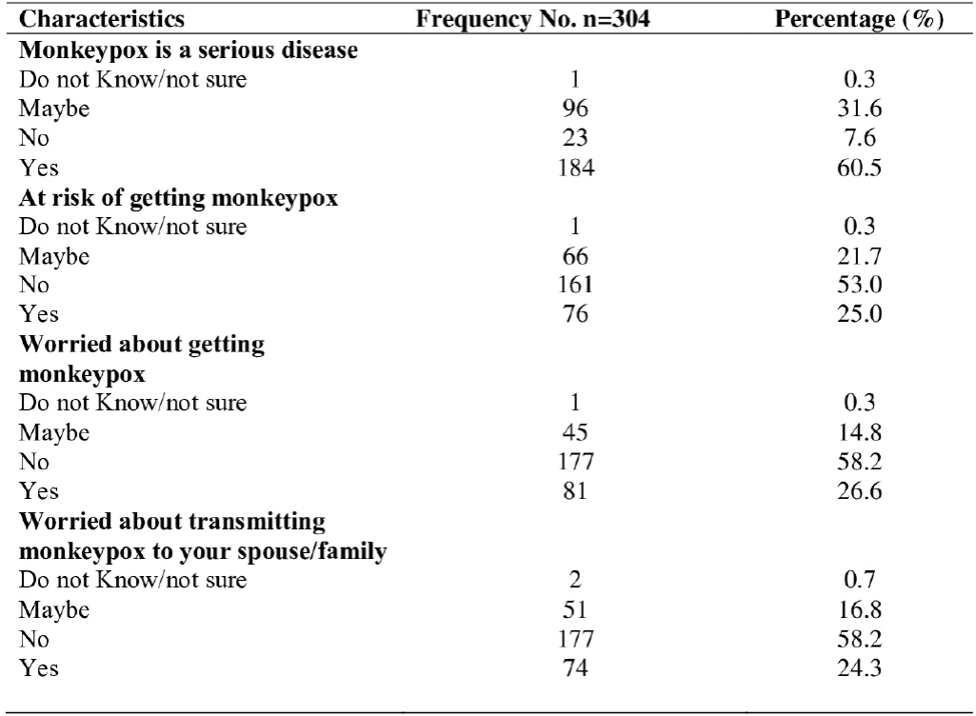
Risk Perception about mpox among people living with HIV and Men who have sex with Men in selected health facilities in Rivers State Nigeria

**Methods:**

This cross-sectional study was carried out using a web-based anonymous Google Forms among PLHIV and MSM at the University of Port Harcourt Teaching Hospital and the Initiative for Advancement of Humanity, respectively in Rivers State, Nigeria from 26 August to 30 September 2024, just before the commencement of mpox vaccines for the first time in Nigeria. Data on sociodemographic characteristics, mpox knowledge and perception, and willingness to receive the mpox vaccine were obtained. Chi-square test was used to assess the relationship between sociodemographic information and participants' mpox knowledge, attitude towards, and willingness to accept the mpox vaccine. Multivariate logistic regression was used to identify determinants of vaccination willingness.

**Figure:**
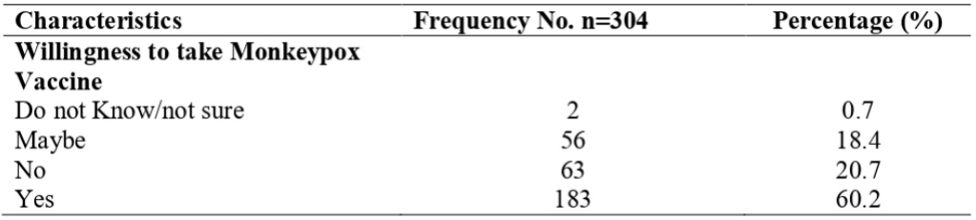
Willingness to accept the Mpox Vaccine

**Figure:**
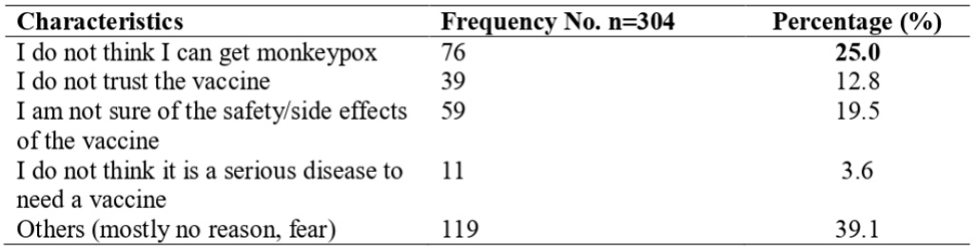
Reasons for unwillingness to accept the Mpox Vaccine

**Results:**

Of 304 participants, the majority were female (59.5% [181]). Slightly more than one-quarter of the respondents had good mpox knowledge (26.3% [n=80]) and self-perceived a high risk of mpox (25.0% [n=75]), while 60.2% (183) indicated willingness to receive the mpox vaccine. Willingness to accept the mpox vaccine was positively associated with younger age and knowledge level. The odds of accepting the mpox vaccine were higher among participants who were less than 25 years (X2 = 9.781; p < 0.007) and those with good knowledge of mpox (X2 = 7.272; p< 0.027). Reasons for hesitancy included low risk perception, concerns regarding vaccine safety, and mistrust of authorities.

**Conclusion:**

Mpox-related knowledge, risk perception, and vaccine acceptance were suboptimal among PLHIV and MSM populations in Rivers State, Nigeria. Vaccine acceptance was influenced by the level of knowledge towards mpox, underscoring the need for targeted risk communication and education on mpox to enhance acceptance of mpox vaccination among high-risk populations in Nigeria.

**Disclosures:**

All Authors: No reported disclosures

